# Supplementary material for: HIVprotI: an integrated web based platform for prediction and design of HIV proteins inhibitors
Source: J Cheminform. 2018 Mar 9;10:12. doi: 10.1186/s13321-018-0266-y (PMC5845081; doi:10.1186/s13321-018-0266-y)
Supplement: Supplementary file 2 — Additional file 2. Source code of HIVProtI web server. [file 13321_2018_266_MOESM2_ESM.zip › HIVprotI_Source-code/hivprotI/weka-3-6-6/documentation.html]

WEKA - Documentation


## WEKA - Documentation

Weka Manual

Package Documentation

#### Online resources

Weka Homepage

WekaWiki (HOWTOs, code snippets,
etc.)

Weka community documentation at Pentaho

Weka on SourceForge.net
